# Demand for Family Planning Satisfied With Modern Methods in Urban Malawi: CHAID Analysis to Identify Predictors and Women Underserved With Family Planning Services

**DOI:** 10.3389/fgwh.2021.652902

**Published:** 2021-05-28

**Authors:** Nurudeen Alhassan, Nyovani Janet Madise

**Affiliations:** African Institute for Development Policy (AFIDEP), Lilongwe, Malawi

**Keywords:** demand for family planning satisfied with modern methods, urban, Malawi, CHAID, underserved women

## Abstract

**Introduction:** Family planning progress under the SDGs is measured with a novel indicator, demand for family planning satisfied with modern methods (mDFPS), which provides a better indication of modern contraceptive coverage than unmet need and contraceptive prevalence rate. Yet, few studies have examined the predictors of mDFPS and the sub-groups of women with unsatisfied mDFPS in urban Saharan Africa. The objective of this study was to examine the predictors of mDFPS in urban Malawi and to identify the sub-groups of urban women underserved with modern contraceptives.

**Methods:** The study analysed data from the 2015–16 Malawi Demographic and Health survey. The sample was comprised of 2,917 women in urban Malawi who had a demand for family planning services. We used a Chi-square (χ^2^) Automatic Interaction Detector (CHAID) model to address the study objectives.

**Results:** The results show that the number of living children a woman had was the most significant predictor of mDFPS. Women with one or more children, who were of Chewa, Lomwe, or Tumbuka ethnic origin and who resided in the central region had the highest mDFPS (87%). On the other hand, women with no children, and who were not exposed to FP information on television, had the lowest mDFPS (41%). Among women in union, ethnicity was the best predictor of mDFPS. Ngoni, Yao, and other ethnic minority women in union who were aged 15–19 and 40 years and above and those who were Catholic, SDA/Baptist, or Muslim had the lowest mDFPS (36%).

**Conclusion:** This study demonstrates significant intra-urban disparities in demand for FP satisfied with modern contraceptives in Malawi. There is a need for policymakers and reproductive health practitioners to recognise these disparities and to prioritise the underserved groups identified in this study.

## Introduction

Modern contraceptive use is effective for preventing unplanned pregnancies and helping individual women and couples to decide freely and responsibly if, when, and how many children they want to have (1, 2). Since the adoption of the Programme of Action of the International Conference on Population and Development (ICPD PoA) in 1994, the provision of safe, effective, and affordable methods of contraception has been an integral part of the efforts to promote sexual and reproductive health and empower women and girls, particularly in Low- and Middle-Income Countries (LMIC) (3). Access to modern contraceptives has also been shown to contribute to poverty reduction through improvement in educational outcomes and economic opportunities for women and girls (4–6). Given these extensive benefits, the global community and many national governments have, over the years, undertaken policy actions and made investments to increase access to family planning (FP) services. These efforts, including the Sustainable Development Goals (SDGs), the FP2020 Initiative, and the Maputo Protocol, have resulted in significant increases in contraceptive use in many parts of the world, with the global contraceptive prevalence rate increasing from 35% in 1970 to 63% in 2017 (1). Even though contraceptive use in Africa has increased steadily, prevalence is still only 36%, which is about half of the global average (1).

To monitor progress in contraceptive coverage, several indicators and measures have been adopted under various global development initiatives including contraceptive prevalence rate (CPR) and unmet need for FP. Contraceptive prevalence rate and unmet need were the main FP indicators used to measure progress in target 5B of the Millennium Development Goals (MDGs), to “achieve, by 2015, universal access to reproductive health.” While these indicators remain useful, progress in FP targets under the SDGs is being measured with a novel indicator, demand for FP satisfied with modern contraceptive methods (mDFPS). This indicator is defined as the proportion of women using modern contraceptives among those in need of contraception (7, 8). Compared to unmet need and contraceptive prevalence, mDFPS provides a better indication of modern contraceptive coverage as the denominator for its estimation is limited to sexually active women of reproductive age in need of contraception (7).

Despite providing a better indication of modern contraceptive coverage and being the main FP indicator for measuring progress in the SDGs, very few studies have examined the predictors of mDFPS within LMICs and the women whose demand for FP is not being satisfied with modern methods (7, 9). Many of the studies on mDFPS have focused on macro-level analysis at the global level, among LMICs, and also at the sub-Saharan African regional level (1, 2, 7, 8, 10). The few studies at the country level have focused on rural areas where the mDFPS coverage is generally low (9). To the best of our knowledge, no study to date has examined mDFPS in an urban setting in sub-Saharan Africa where contraceptive coverage is fairly high relative to rural areas. Yet, such a study will contribute to understanding intra-urban disparities in modern contraceptive coverage and the categories of urban women whose demand for FP is not being satisfied with modern methods. With the majority of Africa's population projected to live in urban areas by 2050 (11), understanding and addressing intra-urban disparities in mDFPS will be critical for managing the adverse effects of having so many urban dwellers. Urban populations are also more diverse in terms of ethnicity, education, wealth, etc. than rural areas; therefore disparities in mDFPS are wide and complex.

This study examines mDFPS in an urban setting in sub-Saharan Africa, i.e., Malawi. The study will help policymakers and reproductive health practitioners in Malawi to identify and prioritise urban women underserved with modern contraceptives, in the spirit of “leaving no one behind.” The study has two specific objectives: (a) examine the predictors of mDFPS in urban Malawi and (b) identify the sub-groups of urban women whose demand for FP is not being satisfied with modern contraceptives.

## Contraceptive Use Context in Urban Malawi

The Republic of Malawi is a landlocked country in southeast Africa, with an estimated population of 17.5 million in 2018 (12). The National Statistical Office (NSO) of Malawi defines urban areas on the basis of concentration of non-agricultural activities such as trading and manufacturing, population density, level of service delivery, and a minimum total population of 5,000 people (13). It is estimated that 16% of the total population of Malawi lives in urban areas, making it one of the least urbanised countries in the world (12, 14). Nevertheless, the country is urbanising rapidly at a rate of 4.2%, higher than the average for sub-Saharan Africa (15). The urban population has been projected to reach 12.4 million by 2050, from 2.7 million in 2014 (15). Malawi's urban population is concentrated in four major cities—Blantyre, Lilongwe, Mzuzu, and Zomba—which account for 75% of the urban population (12). The rapid urbanisation in Malawi is mainly driven by high fertility in urban areas and rural-urban migration. For example, the mean number of children ever born to women age 40–49 years in urban areas is 4.6 children (16).

Even though urban infrastructure and service provision in Malawi is less developed compared to countries such as Kenya and South Africa, the urban population still has better access to social services relative to their rural counterparts. For example, 42% of the urban population has access to electricity compared to just 4% in rural areas (17). Urban residents also have better access to healthcare services including reproductive health compared to rural residents. For instance, while the urban population makes up 16% of the total population, 40% of private health facilities are located there and a further 13% in peri-urban areas, with just 47% in rural areas (18). These rural-urban disparities in access to critical social services especially healthcare translate into an urban advantage in access to modern contraceptives. Approximately 78% of demand for FP among married women in urban areas is satisfied with modern methods compared to 74% in rural areas (16). However, this apparent urban advantage represents aggregate levels that mask intra-urban differentials. Yet, little is known of the disparities in demand for FP satisfied with modern methods in urban Malawi. This study, therefore, sought to fill this knowledge gap and to help policymakers and reproductive health practitioners to identify the predictors of mDFPS and the sub-groups of urban women whose demand for FP remains unsatisfied with effective modern methods.

## Methods

### Study Design and Data

The current study analysed data from the 2015–16 Malawi Demographic and Health Survey (16). This was a nationally representative survey conducted by NSO in collaboration with the DHS Programme. A two-stage stratified sampling technique was used to select a total of 25,146 eligible women for the survey. A detailed description of the sampling procedure for the survey is available in the final report of the survey (16). Of the 25,146 women eligible to be interviewed, 24,562 were actually interviewed. This comprised 5,247 women in urban areas and 19,315 in rural areas. In this paper, we analysed data from a sub-sample of 2,917 urban women who had a demand for family planning. This sample was made of 2,371 women currently in union (married/living with a partner) and 546 unmarried (never married/formerly married) women who had sex 1 month preceding the survey. For the bivariate analysis, we weighted the data to take into account unequal sampling probabilities, and we also took into account the complexity (clustering and stratification) of the DHS sampling design. In DHS surveys, the sample is usually selected with unequal probability to expand the number of cases available for certain areas or population sub-groups for which data is needed. Weights, therefore, need to be applied when tabulations are made to produce an accurate representation.

### Study Variables

The dependent variable for this study was the demand for family planning satisfied with modern contraceptives (mDFPS). This variable (mDFPS) was calculated based on the 2012 update of the indicator definition by MEASURE DHS (19). The numerator for mDFPS was the number of reproductive age women, who were either married or unmarried but reported sexual intercourse in the 1 month preceding the survey, and who were currently using any modern contraceptive method. The denominator was the total number of women in need of contraception. The women in need of contraception included the following:

a. Fecund women who wanted the next child after 2 years
b. Fecund women who wanted another child but were undecided on the timing or were undecided if they want another child
c. Fecund women who wanted no more children
d. Pregnant women who wanted the pregnancy later or did not want it at all
e. Post-partum amenorrheic women who wanted their last birth later or did not want it at all

Women were classified as infecund and therefore did not need contraceptive if they fell into the following categories:

a. Married for 5 or more years, had no children in the past 5 years, and never used contraception
b. Reported that they could not get pregnant
c. Reported that they were menopausal/hysterectomy or never menstruated
d. Had last period more than 6 months and are not post-partum amenorrheic

Modern contraceptive methods in this study included contraceptive pills, intrauterine devices (IUD), injectables, condoms (male and female), sterilisation (male and female), implants, and emergency contraceptives. It is important to note that while Standard Days Method and Lactational Amenorrhea are considered modern methods by the World Health Organisation (WHO) and the Demographic and Health Survey (DHS), we excluded these two methods from our definition of modern methods in this study. We limited our definition of modern methods to biomedical methods that are actively being promoted in Malawi's family policies such as in the Costed Implementation Plan (20). Because the use of these two methods is very negligible (<0.5%) in the sample, excluding them from the definition of modern methods did not affect our measurement and analysis in any way.

We classified the dependent variable into two categories (Yes/No): women using biomedical modern contraceptive methods among those in need of contraception were considered to have a demand for FP satisfied with modern methods (Yes) while those not using any method and those using traditional methods including periodic abstinence, withdrawal, standard days method and lactational amenorrhea among those in need of contraception were considered to have an unsatisfied mDFPS (No).

The predictor variables included socio-demographic and reproductive characteristics of the women as well as characteristics of their partners for those in union (Tables 1, 2). These characteristics included age, number of living children, highest educational attainment, wealth quintile, religious affiliation, ethnicity, region of residence, employment status, and exposure to family planning information on radio, TV, in newspapers/magazines, or at health facilities. For the women in union, we also included as predictors the age at first marriage, age difference with their partners, partner's highest education, partner's employment status, and the partner's fertility preference.

**Table 1:** Socio-demographic characteristics of women with demand for FP in Malawi.

**Socioeconomic and demographic characteristics**	**Percent (%)**		**Urban number**
	**National weighted**	**Urban weighted**	
**Demand for FP satisfied with modern methods**			
Yes	73.9	76.2	2,229
No	26.1	23.8	688
**Age groups**			
15–19	9.2	7.5	242
20–24	21.6	19.1	574
25–29	19.3	22.0	648
30–34	19.1	21.6	573
35–39	15.4	15.5	452
40–44	9.5	9.7	273
45–49	6.0	4.7	155
**Highest education**			
No education	13.1	4.3	140
Primary	64.3	41.8	1,295
Secondary	20.1	43.1	1,233
Higher	2.5	10.8	249
**Wealth quintile**			
Poorest	19.8	19.5	708
Poorer	20.6	20.0	567
Middle	20.5	20.8	550
Richer	19.9	20.3	571
Richest	19.2	19.3	521
**Religion**			
Catholic	18.0	17.1	483
CCAP	16.8	21.6	592
Anglican	2.5	3.2	140
Seventh day adventist/baptist	6.6	8.4	249
Other christian	43.7	40.0	1,121
Muslim	11.7	9.2	320
No religion	0.6	0.4	11
Other	0.1	0.0	1
**Ethnicity**			
Chewa	35.8	24.5	715
Tumbuka	9.1	10.4	339
Lomwe	19.5	20.8	543
Yao	12.4	13.4	338
Ngoni	11.5	17.3	453
Other	11.7	13.7	529
**Region**			
Northern	11.8	10.3	604
Central	43.4	45.4	1,011
Southern	44.9	44.4	1,302
**Employment status**			
Employed	68.4	60.4	1,806
Unemployed	31.6	39.6	1,111
**Marital status**			
Never in union	5.9	9.3	276
Currently married	83.8	80.9	2,371
Formerly in union	10.4	9.8	270
**Number of living children**			
No child	5.4	7.3	226
1–2 children	36.9	47.2	1,303
3–4 children	33.1	32.6	965
5+ children	24.6	12.8	423
**Exposure to FP information on radio**			
Yes	42.9	57.7	1,764
No	57.1	42.3	1,153
**FP information on TV**			
Yes	10.0	31.0	863
No	90.0	69.0	2,054
**FP information in newspaper/magazine**			
Yes	8.8	20.1	586
No	91.2	79.9	2,331
**FP information by text messages**			
Yes	5.7	14.0	360
No	94.3	86.0	2,557
**FP information in health facility**			
Yes	23.5	23.5	912
No	39.2	39.2	1,103
Did not visit a health facility	37.3	37.2	902

*Source: Malawi Demographic and Health Survey, 2015–16*.

**Table 2:** Socio-demographic characteristics of married women with demand for FP in Malawi.

**Socioeconomic and demographic characteristics**	**Percent (%)**		**Urban number**
	**National weighted**	**Urban weighted**	
**Demand for FP satisfied with modern methods**			
Yes	74.5	77.4	1,827
No	25.5	22.6	544
**Age groups**			
15–19	5.9	3.3	101
20–24	21.5	18.2	431
25–29	20.4	23.9	568
30–34	20.1	22.5	496
35–39	15.8	16.8	405
40–44	10.1	10.5	239
45–49	6.2	4.9	131
**Highest education**			
No education	13.5	4.3	120
Primary	65.1	42.0	1,070
Secondary	19.1	43.1	982
Higher	2.3	10.6	199
**Wealth quintile**			
Poorest	18.6	19.8	575
Poorer	21.0	20.7	468
Middle	20.9	21.7	444
Richer	20.4	20.4	462
Richest	19.2	17.4	422
**Religion**			
Catholic	17.5	16.1	383
CCAP	17.1	22.3	491
Anglican	2.4	2.7	100
Seventh day adventist (SDA)/baptist	6.8	8.8	208
Other christian	44.1	40.0	916
Muslim	11.6	9.6	263
No religion	0.5	0.5	9
Other	0.1	0.0	1
**Ethnicity**			
Chewa	36.4	23.7	587
Tumbuka	9.7	10.9	289
Lomwe	18.5	20.6	422
Yao	12.0	13.6	264
Ngoni	11.5	17.2	382
Other	11.8	14.1	427
**Region**			
Northern	12.5	11.0	508
Central	44.3	45.7	839
Southern	43.2	43.4	1,024
**Employment status**			
Employed	68.8	61.0	1,501
Unemployed	31.2	39.0	870
**Age at first marriage**			
<18 years	50.8	37.0	952
18–24 years	45.3	54.1	1,261
25+ years	3.9	8.8	158
**Number of living children**			
No child	1.9	1.5	49
1–2 children	37.3	49.3	1,085
3–4 children	34.6	35.9	869
5+ children	26.2	13.3	368
**FP information on radio**			
Yes	55.9	59.3	1,479
No	44.1	40.7	892
**FP information on TV**			
Yes	10.0	31.2	718
No	90.0	68.8	1,653
**FP information in newspaper/magazine**			
Yes	8.3	19.5	473
No	91.7	80.5	1,898
**FP information by text messages**			
Yes	5.7	14.5	305
No	94.3	85.5	2,066
**FP information in health facility**			
Yes	34.3	25.5	798
No	35.9	40.3	903
Did not visit a health facility	29.8	34.2	670
**Age difference**			
1–4 years	43.4	41.0	973
5–9 years	33.8	38.6	890
10/more years	14.7	14.5	380
Same age	4.0	3.2	62
Wife older	4.1	2.8	66
**Husband's education**			
No education	9.3	2.3	72
Primary	54.2	26.1	693
Secondary	30.1	52.7	1,188
Higher	5.3	18.2	393
Don't know	1.1	0.7	25
**Husband's employment**			
Unemployed	8.6	3.2	112
Employed	90.6	96.3	2,240
Don't know	0.8	0.6	19
**Husband's fertility desire**			
Both want same	59.7	65.5	1,317
Husband wants more	18.0	15.6	354
Husband wants fewer	9.9	9.8	155
Don't know	12.4	9.1	208

*Source: Malawi Demographic and Health Survey, 2015–16*.

### Statistical Analysis

We used descriptive statistics (percentages) to assess the dependent (mDFPS) and predictor variables that were used for the Pearson's χ^2^ and the χ^2^ Automatic Interaction Detector (CHAID) analyses. Pearson's χ^2^ test was performed to examine the associations between mDFPS and key background characteristics of the women. CHAID analysis was then used to examine the significant predictors of mDFPS, and to identify the subgroups of urban women whose demand for FP remains unsatisfied with effective modern methods. CHAID is a methodological approach that is rarely used in family planning research. However, as a predictive model that outlines variables that have the strongest impact on group differentiation, CHAID is more sophisticated than conventional logistic regression which is often used in family planning studies. CHAID analysis also allows for the identification of characteristics that define groups and sub-groups (21, 22). It is these advantages of CHAID that informed the decision to use it, as it is more appropriate for the objectives of this study.

CHAID is a non-parametric and non-linear decision tree algorithm that makes no distributional assumption on outliers, collinearities, heteroscedasticity, or distributional error structures. The dependent variable in CHAID analysis can be nominal, ordinal, or interval. Unlike regression models where statistical effects are fitted simultaneously, CHAID uses a sequential fitting method where the effects of later statistical tests are dependent on earlier ones. At each stage in this sequence, the predictor variable(s) with the strongest association to the dependent variable is selected. The process continues until all significant predictors have been identified. The predictor variable with the strongest association to the dependent variable is usually the first branch in a tree-like model, with leaves representing categories that are significantly different relative to the dependent variable. The dataset is then further categorised into subgroups according to this first predictor variable, with the most significant combination of variables selected.

The output of a CHAID prediction model is a hierarchical tree-like diagram, which consists of several levels of branches referred to as nodes: root node, parent node, child nodes, and terminal nodes. Node splitting is obtained by selecting and using the predictor variable with the most significant *p*-value as a node separator in each stage of the analysis. This is done by comparing *p*-values of each predictor variable in the previous stage. This process of node splitting continues until there is no predictor variable with the most significant *p*-value (21). The root node (node 0) is the dependent variable, in this study mDFPS. Parent nodes are in the upper level of the hierarchical structure compared to nodes in the subsequent lower levels. The lower level nodes are referred to as child nodes while terminal nodes are the last categories of the CHAID analysis tree. Terminal nodes do not have child nodes. Each node provides the number and percentage of people in a selected category or subgroup. Chi-square statistics and *p*-values associated with a statistical test at each level of the CHAID model are also calculated and presented in the tree-structured diagram. The *p*-values in this analysis were adjusted using a Bonferroni correction, meaning the level of significance has been corrected for the several tests of independence performed simultaneously between the predictor variables and the outcome (21). Using Bonferroni correction to adjust *p*-values reduces the likelihood of committing a type I error. All analyses for this paper were conducted using SPSS V.20.

## Results

### Description of Women in Need of Contraception in Malawi, Nationally and in Urban Areas

Table 1 presents the characteristics of all women with a demand for contraception nationally and in urban areas of Malawi. Nationally, ~74% of women with demand for contraception were using modern methods. Demand for FP satisfied with modern methods was slightly higher in urban areas, with a little over three-quarters of the urban women using modern methods. The mean age of women with demand for contraception nationwide and in urban areas was similar: 30 years. The level of education of urban women with demand for FP was higher than the level nationally, with 4% of the urban women having no education compared to 13% nationwide. However, the percentage of unemployed women in urban areas (39.6%) was higher than the percentage nationwide (31.6%). The percentage of women with no children in urban areas was higher than the percentage nationally. About 13% of the urban women had five or more children compared to a quarter of all women in Malawi. The proportion of urban women exposed to FP information on radio, TV, in newspapers/magazines, or at health facilities was higher in urban areas than the proportion nationwide.

Given that marriage and sexual unions affect contraceptive use in various ways including through spousal influence, we anticipated that the predictors of mDFPS may be different for women in union. We therefore also conducted analyses with the sub-sample of women in union. Table 2 presents the characteristics of women in sexual unions with demand for contraception nationally and in urban areas. Nationwide, approximately three-quarters of married women with demand for contraception were using modern methods. The percentage of married women in urban areas with demand for contraception using modern methods was slightly higher (77%) than nationally. More than half (51%) of the women in union nationally entered into their first union as children (below age 18 years) while 37% of the urban women in union were married as children. Approximately 60% of women nationwide had the same fertility preference as their partners compared to two-thirds of urban women. As high as 12% of married women with demand for contraception nationally did not know the fertility preference of their partners. Among married women in urban areas, that figure was 9%.

### Factors Associated With Demand for FP Satisfied With Modern Methods in Urban Malawi

Table 3 presents the factors associated with demand for FP satisfied with modern methods among all women in urban Malawi. The results showed that seven factors were significantly associated with mDFPS; age, ethnicity, region, marital status, employment status, number of living children, and exposure to FP information in a health facility.

**Table 3:** Association between mDFPS and socio-demographic and reproductive characteristics of all urban women in Malawi.

**Background characteristics**	**Demand for FP satisfied with modern contraceptives**			
**Age**	**Yes (%)**	**No (%)**	**χ^2^**	***p*-value**
15–19	62.5	37.5		
20–24	78.9	21.1		
25–29	79.2	20.8		
30–34	78.7	21.3	40.58	0.023
35–39	76.8	23.2		
40–44	68.1	31.9		
45–49	77.0	23.0		
**Highest education**				
No education	79.2	20.8		
Primary	79.4	20.6	15.09	0.168
Secondary	74.3	25.7		
Higher	70.8	29.2		
**Wealth quintile**				
Poorest	77.2	22.8		
Poorer	77.5	22.5		
Middle	77.3	22.7	4.15	0.714
Richer	73.2	26.8		
Richest	76.1	23.9		
**Religion**				
Catholic	73.3	26.7		
CCAP	79.4	20.6		
Anglican	80.3	19.7	21.59	0.136
SDA/Baptist	70.8	29.2		
Other Christian	78.2	21.8		
Muslim	68.8	31.2		
**Ethnicity**				
Chewa	82.3	17.7		
Tumbuka	79.4	20.6		
Lomwe	79.4	20.6	41.72	0.011
Yao	70.2	29.8		
Ngoni	70.7	29.3		
Other	71.2	28.8		
**Region**				
Northern	69.8	30.2		
Central	79.2	20.8	14.76	0.006
Southern	74.7	25.3		
**Marital status**				
Never in union	59.0	41.0		
Currently married	77.4	22.6	54.68	0.000
Formerly married	83.5	16.5		
**Employment status**				
Employed	78.8	21.2	16.67	0.007
Unemployed	72.3	27.7		
**Number of living children**				
No child	48.9	51.1		
1–2 children	78.7	21.3	95.33	0.000
3–4 children	78.5	21.5		
5+ children	77.0	23.0		
**FP information on radio**				
Yes	74.6	25.5	6.24	0.084
No	78.5	21.5		
**FP information on TV**				
Yes	76.5	23.5	0.05	0.881
No	76.1	23.9		
**FP information in newspaper**				
Yes	77.0	23.0	0.22	0.757
No	76.1	23.9		
**FP information via text message**				
Yes	72.1	27.9	4.48	0.227
No	76.9	23.1		
**FP information in health facility**				
Yes	73.5	26.5		
No	80.4	19.6	18.10	0.030
Did not visit facility	73.6	26.4		

Demand for FP satisfied with modern methods was high among women aged 25–29 years (79%) and low among women aged 15–19 years (63%) and those aged 40–44 years (68%). With regards to ethnicity, mDFPS was higher among Chewa (82%), Lomwe (79%), and Tumbuka (79%) women and lower among Yao (70%), Ngoni (71%), and other minority women (71%). mDFPS was higher among women in the central region (79%) and lower in the northern region (70%). Women who had never been in a marital union (59%) had a lower mDFPS compared with those that were currently in union and those formerly married. Furthermore, 79% of women in employment had their demand for FP satisfied with modern methods compared with 72% among unemployed women. Moreover, mDFPS was highest among women with one or two children (79%) and very low among those with no children (49%). Contrary to expectation, mDFPS was higher among women that did not receive FP information in a health facility and lower among those that received such information in a health facility and those that did not visit health a facility.

### Results of CHAID Analysis for All Urban Women With Demand for Contraception

**Figure 1:**
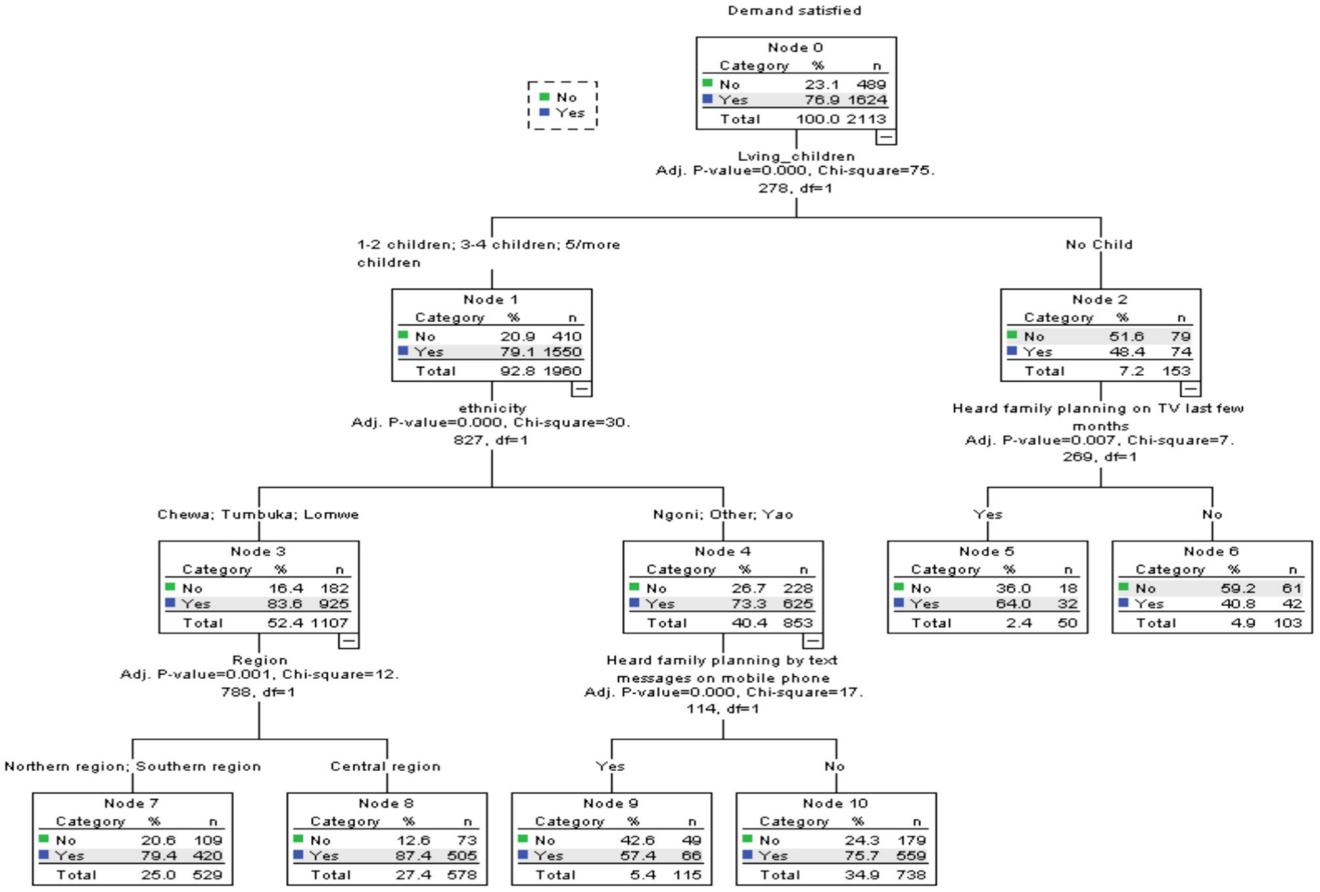
Decision tree diagram for all urban women with demand for modern methods in Malawi.

Figure 1 presents results of the CHAID analysis for all urban women with a demand for contraception. The results showed that the number of living children a woman had was the most significant predictor of mDFPS (*X*^2^ = 75.56, *p* = 0.000). This variable was spilt into two nodes. Node 1 consisted of women with one or more living child, while node 2 was made of women with no children.

#### Node 1: Women With One or More Children

For this subgroup of women who constituted ~93% of the sample, ethnicity was the strongest predictor of mDFPS (*X*^2^ = 30.60, *p* = 0.000). About 79% of the demand for FP among these women was satisfied with modern methods. The next most important variable for this group of women, ethnicity, was spilt into subgroups: Chewa, Tumbuka, and Lomwe (node 3) and Ngoni, Yao, and Other (node 4). Women in the Chewa, Tumbuka, and Lomwe subgroup (node 3) had 84% of their demand for FP satisfied with modern contraceptives. However, those in the Ngoni, Yao, and other ethnic groups had 73% of their demand for FP satisfied with modern methods. For women in the Chewa, Tumbuka, and Lomwe subgroup, region of residence was the next best predictor of mDFPS (*X*^2^ = 13.30, *p* = 0.001). Women in the northern and southern regions (node 7) had 79% of their FP demand satisfied with modern methods compared to 87% among those in the central region.

Among Ngoni, Yao, and women of other minority ethnic groups, exposure to FP information via text messages on phone was the strongest predictor of mDFPS (*X*^2^ = 16.04, *p* = 0.000). Only about 58% of the women who received FP information via text messages on their phones (node 9) had their demand for FP satisfied with modern methods compared to ~76% of those that did not (node 10).

#### Node 2: Women With No Children

Only 48% of the women with no children had their demand for FP satisfied with modern contraceptives. Among these women, exposure to FP information on TV was the best predictor of demand for FP satisfied with modern methods (*X*^2^ = 7.27, *p* = 0.007). Close to two-thirds (64%) of the women who watched FP information/messages on TV in the last few months had their demand for FP satisfied with modern contraceptives compared to about just 41% of those that did not. Table 4 provides summary information of the specifications used to build the CHAID model for the full sample.

**Table 4:** Summary information of the specifications used to build the CHAID model for all urban women.

**Model Components**	**Model specification**	**Results**
Dependent variable	Demand for FP satisfied with modern methods	% of women with demand satisfied = 76.9
Independent variables	Age, highest education, wealth quintile, religion, ethnicity, region, employment, marital status, exposure to FP information on radio, exposure to FP information on TV, exposure to FP information in newspapers/magazines, exposure to FP information by text messages on mobile phone, exposure to FP information in health facility, number of living children	*Number of living children*, ethnicity, region of residence, exposure to FP information on TV, exposure to FP information by text messages on mobile phone.
Maximum tree depth	3	3
Minimum cases in parent node	100	100
Minimum cases in child node	50	50
Number of nodes	–	11
Number of terminal nodes	–	6
Overall predicted correct percentage		77.8

### Results of CHAID Analysis for Urban Women in Union With Demand for Contraception

**Figure 2:**
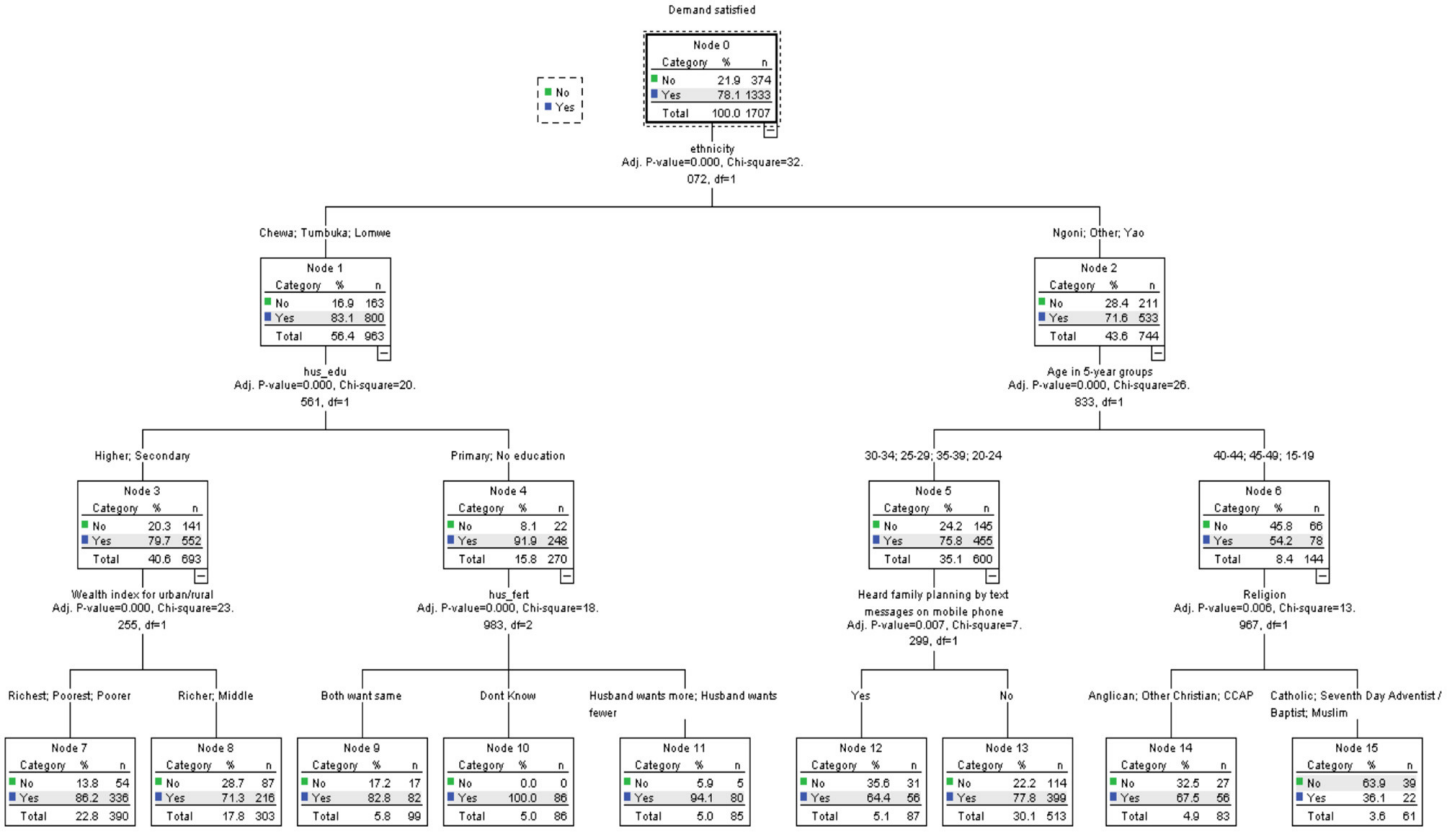
Decision tree diagram for married women in urban areas with demand for modern methods in Malawi.

The results of the CHAID analysis for urban women in union with demand for contraception are presented in Figure 2. The results showed ethnicity as the most significant predictor of mDFPS among urban women in union (*X*^2^ = 32.07, *p* = 0.000). Chewa, Lomwe, and Tumbuka (node 1) women in union had 83% of their demand for FP satisfied with modern methods compared to about 72% among Ngoni, Yao, and women of other minority ethnic groups such as Nkhonde and Sena (node 2).

#### Node 1: Chewa, Lomwe, and Tumbuka Women in Union

This sub-group of women constituted 56% of the total sample of women in union. Among these women, the highest educational attainment of their partner was the best predictor of mDFPS (*X*^2^ = 20.56, *p* = 0.000). The educational attainment of their partners was further split into two sub-groups: women with partners of secondary and higher education (node 3), and those with partners of no education and primary education (node 4). Contrary to expectation, as high as 92% of women whose partners had no education or primary education had their demand for FP satisfied with modern contraceptives compared to about 80% of those whose partners had secondary and higher education.

Among women whose partners had secondary or higher education, the household wealth quintile was the best predictor of mDFPS (*X*^2^ = 23.26, *p* = 0.000). In this sub-group, women in the richest, poorer, and poorest (node 7) households had a demand for FP satisfied with modern methods (86%) 15% higher than those in the richer and middle households (71%). For the women whose partners had no education or primary education (node 4), the fertility preference of the couple was the most significant predictor of mDFPS (*X*^2^ = 18.93, *p* = 0.000). Women who had the same fertility preference as their partners had about 83% of their demand for FP satisfied with modern methods while women whose husbands wanted more and those whose husbands wanted fewer children had 94% of that demand satisfied. Unexpectedly, all the women (100%) who did not know the fertility preference of their partners had their demand for FP satisfied with modern methods.

#### Node 2: Ngoni, Yao, and Other Women in Union

As indicated previously, about 72% of Ngoni, Yao, and other women of minority ethnic groups in union had their demand for FP satisfied with modern methods. Among these women, age was the most significant predictor of mDFPS (*X*^2^ = 26.83, *p* = 0.000). Age was further split into two sub-groups: 20–24, 25–29, 30–34, 35–39 (node 5) and 15–19, 40–44, 45–49 (node 6). The mDFPS among women aged 20–24, 25–29, 30–34, and 35–39 years was about 76%. This reduced significantly to 54% among women aged 15–19, 40–44, and 45–49 years.

For the women aged 20–24, 25–29, 30–34, and 35–39 years, the most significant predictor of mDFPS was exposure to FP information through text messages on phone (*X*^2^ = 7.30, *p* = 0.007). About two-thirds (64%) of the women who received FP information via text messages had their demand for FP satisfied with modern methods compared to more than three-quarters of those that did not. Among the women aged 15–19, 40–44, and 45–49 years, religious affiliation was the best predictor of mDFPS (*X*^2^ = 13.97, *p* = 0.006). Approximately 68% of Anglican, CCAP and Other Christian women had their demand for FP satisfied with modern methods compared to just 36% among Catholic, SDA, and Muslim women. Table 5 provides summary information of the specifications used to build the CHAID model for women in union.

**Table 5:** Summary information of the specifications used to build the CHAID model for urban women in union.

**Model components**	**Model specification**	**Results**
Dependent variable	Demand for FP satisfied with modern methods	% of women with satisfied demand = 78.1
Independent variables	Age, highest education, wealth quintile, religion, ethnicity, region, employment, marital status, exposure to FP information on radio, exposure to FP information on TV, exposure to FP information in newspapers/magazines, exposure to FP information by text messages on mobile phone, exposure to FP information in health facility and number of living children, age at marriage, age difference between partners, husband education, husband employment, husband fertility preference	*Ethnicity*, husband education, wealth quintile, husband's fertility preference, age, exposure to FP information by text messages on mobile phone, religion
Maximum tree depth	3	3
Minimum cases in parent node	100	100
Minimum cases in child node	50	50
Number of nodes	–	16
Number of terminal nodes	–	9
Overall predicted correct percentage		79.1

## Discussion

The objective of this study was to examine the predictors of demand for FP satisfied with modern methods (mDFPS) in urban Malawi and to identify the sub-groups of urban women underserved with modern contraceptives. The study analysed data from the 2015–16 Malawi DHS data, using a CHAID decision tree analytic technique. The results for the full sample showed that demand for FP satisfied with modern methods was higher among women with children, Chewa/Tumbuka/Lomwe women, and women residing in the central region. The least demand satisfied with modern methods was among women with no children, and who had not heard FP advertising on television.

The most significant predictor of demand for FP satisfied with modern methods was the number of living children women had. The women with no children had a lower demand satisfied with modern methods relative to those with children. This finding is consistent with the results of previous studies conducted in sub-Saharan Africa and LMICs (23, 24). One study in Zimbabwe found that women with no children were eight times less likely to use modern contraceptives compared to those with one or more children (24). A plausible explanation of the finding in this study is that the desire to postpone first birth is weakly held among women with no children. Thus, the motivation to use modern contraceptives to satisfy their demand for FP is low. It is also likely that most of the women with no children are younger, and therefore face several barriers to accessing modern contraceptives. Encouraging women to postpone first birth and addressing both the demand and supply factors that inhibit women with no children from using modern contraceptives need to be prioritised if Malawi is to close the mDFPS gap between these women and their peers with one or more children.

The results also showed ethnicity as a significant predictor of mDFPS, and ethnicity was significant for women with one or more children. Chewa, Lomwe, and Tumbuka women who had at least one living child had a higher mDFPS than Yao, Ngoni, and other ethnic minority women. Yaos ascribe to matrilineal descent and are predominantly Muslim, with conservative cultural norms that value sexual initiation ceremonies, early marriages, and large families (25). With regards to the Ngoni, patrilineal descent where children are affiliated to their fathers' kin group is a major cultural feature. It is known that women in patrilineal descent systems have limited autonomy in decision making regarding childbearing (26). The above cultural norms probably inhibit modern contraceptive use among Yao and Ngoni women. Women of minority ethnic groups such as the Nkhonde and Sena also probably face difficulties in accessing FP services due to language barriers or discrimination by contraceptive service providers. There is the need for further studies to investigate cultural norms, beliefs and other factors that constrain modern contraceptive use among Yao, Ngoni and other ethnic minority women in urban Malawi.

The analysis further showed region of residence as the most significant predictor of mDFPS among Chewa, Lomwe, and Tumbuka women. Women residing in the central region had a significantly higher mDFPS than those in the southern and northern regions. A plausible explanation of this finding is the low concentration of health facilities especially facilities that offer reproductive services in the northern and southern regions. For example, one study that mapped private health facilities, which are crucial for expanding access to FP services, in Malawi found that only 14% of private facilities with nurse midwives were located in the northern and southern regions, respectively, compared to 71% in the central region. Astonishingly, there were no private mobile clinics in the northern region and only 33% of such facilities were in the southern region compared to 67% in the central region (18).

Exposure to FP information via text message(s) on phone was the most significant predictor of mDFPS among Ngoni, Yao, and women of other minority ethnic groups. Unexpectedly, it was observed that the women that were exposed to FP information via text messages had a lower mDFPS than those that were not. Specifically, about 58% of the Ngoni, Yao, and women of other ethnic groups that had accessed FP information via text messages on phone had their demand for FP satisfied with modern methods compared to ~76% of those that did not. Even though FP programmes are increasingly taking advantage of the ubiquity of mobile phones in sub-Saharan Africa to deliver contraceptive information via text messages to women and couples, there is little evidence of the effect of such interventions on contraceptive uptake. One study that evaluated the impact of a mobile reproductive health platform in Kenya (m4RH) which delivered contraceptive information via text messages found an increase in m4RH consumers' contraceptive knowledge, but there was no increase in contraceptive use among them (27). While we are not sure of the source(s) of the text messages delivered to the women in the current study, it is possible that these messages reinforce misconceptions about FP, and therefore discourage modern contraceptive use. It is known that the emergence of social media platforms, including WhatsApp and Facebook messenger, allow for the easy circulation of unreliable information from dubious sources including information on FP. It is also possible that this counterintuitive finding is a spurious one due to the omission of other important variables in the analysis, a common pitfall of CHAID and other multivariate analytic techniques (28). It is likely that most women that access FP information via text messages are those with a weak motivation to use modern methods due to fear of side effects. There is a need for further studies to investigate the content, sources, and effect of such messages in Malawi and Africa at large.

As shown in the results, women with no children are severely underserved with FP services, with only 48% of them having their demand for FP satisfied with modern methods. Among these women, exposure to FP information on TV was the best predictor of mDFPS. Approximately two-thirds of the women with no children who were exposed to FP messages on TV had their demand for FP satisfied with modern methods compared to 4 in 10 of those that were not exposed to FP messages on TV. Contrary to the results on the effect of exposure to FP information via text messages, this finding suggests that viewing FP messages on TV improves the chances of using modern contraceptives. Television is a credible source of information in the Malawian context, thus FP information on TV is more likely to be accurate, create awareness, increase knowledge and induce contraceptive use.

For the women in union, the results showed that demand for FP satisfied with modern methods was highest among Chewa, Tumbuka, and Lomwe women whose partners had primary or no education, and who did not know the fertility preference of their partners. The least demand satisfied with modern methods was among Ngoni, Yao, and other ethnic minority women who were aged 15–19 and 40 years and above and were Catholic, SDA/Baptist, or Muslim.

Overall, ethnicity was the strongest predictor of mDFPS among women in union. Similar to the results for the full sample, Chewa, Lomwe, and Tumbuka women in union had a higher mDFPS than Ngoni, Yao, and women of other minority ethnic groups. The fact that ethnicity was the most significant predictor of mDFPS among women in union suggests that socio-cultural norms that influence women's contraceptive use behaviour are at their strongest in sexual unions. Efforts to address sociocultural norms inhibiting women's use of modern contraceptives in urban areas in Malawi need to prioritise those in union, especially Ngoni, Yao, and women of other minority ethnic groups.

Contrary to expectation, the results showed that Chewa, Lomwe, and Tumbuka women whose partners had no education or primary education had a significantly higher mDFPS than those with partners of secondary and higher education. Even though previous studies in LMICs show a strong positive association between educational attainment and modern contraceptive use, there is evidence that better-educated women in urban sub-Saharan Africa consistently report higher use of traditional methods than their less-educated peers (29). While most of the studies reporting high traditional method use among better educated urban women do not include the educational attainment of their partners, it is likely that partners with secondary and higher education disapprove of modern methods due to side effects. It is also possible that FP programmes in urban Malawi are focusing less on couples of higher socio-economic status including women with partners of secondary and higher education because of the wrong assumption that such women are already contracepting or have fewer barriers to accessing modern contraceptive methods.

Furthermore, the study found that household wealth status was the most significant predictor of mDFPS among women with partners of secondary and higher education. Counterintuitively, women in the poorer and poorest categories together with those in the richest category had an mDFPS that was 15% higher than their peers in the richer and middle categories. The bivariate analysis (Table 3) showed no statistically significant difference between rich and poor women in mDFPS, even though the percentages in the poorer and poorest categories were higher than richer and richest. Research in the slums of Kenya has shown that the gap between rich and poor women in terms of access to modern contraceptives has narrowed significantly to a point where there is virtually no difference (30). The narrowing of this gap in Kenya is explained by the increasing focus of family planning and reproductive health programmes on poor, marginalised, and hard-to-reach urban women. Malawi's Family Planning Costed Implementation Plan also identifies urban poor women as one of special sub-groups to focus on increased uptake of modern methods (20). It is possible that this programmatic focus on urban poor has resulted in them having a higher mDFPS. Furthermore, it is important to note that other factors including the motivation for contraceptive use and fear of side effects which were not included in this study determine women's capacity to satisfy their demand for FP with modern methods. It is therefore possible that the omission of these key factors has resulted in a spurious relationship between household wealth and mDFPS. There is a need for further studies to investigate the counterintuitive relationships between socio-economic status and mDFPS observed in this study.

For the women whose partners had no education or primary education, the fertility preference of the couple was the most significant predictor of mDFPS. Women who had the same fertility preference as their partners had a lower mDFPS relative to those whose partners wanted more or fewer children and those who did not know the fertility preference of their partners. Research on fertility preference in Malawi shows that couples with the same fertility preference are those who tend to want a child in the next 3 years (31). This suggests that the women reporting the same fertility preference as their partners in this study are those wishing to postpone or space pregnancy/childbirth but not to stop. In such instances, women are typically less likely to use modern contraceptives to satisfy their demand (31, 32). Interestingly, we also found that all the women that did not know the fertility preference of their partners had their demand for FP satisfied with modern methods. This suggests that when women do not know the fertility preference of their partners they assume a lack of or less opposition from those partners on modern contraceptive use and are therefore likely to have their demand for FP satisfied with modern methods.

As shown in the results, Ngoni, Yao, and other ethnic minority women in union who were aged between 20 and 39 years, the prime reproductive childbearing ages had a significantly higher mDFPS than those aged 15–19, 40–44, and 45–49 years. In general, reproductive health and family planning services in most sub-Saharan African countries including Malawi are not adequately oriented toward meeting the needs of adolescents. Thus, adolescents disproportionately face many barriers in accessing modern contraceptives including stigma, cost of services, and lack of adequate knowledge. The finding in this study is therefore consistent with the findings of previous studies in the sub-region.

With respect to relatively older women, those aged 40 years and above, studies show that they are among those with the highest demand for FP for stopping childbearing (33). It is likely that most urban women in Malawi aged 40 years and above have already attained their desired fertility, and would therefore like to use long-acting and permanent methods (LAPMs) to stop childbearing. Yet, short-term methods such as pills and injectables which are prone to discontinuation and may not satisfy the particular needs of these women are still the dominant modern contraceptive methods in Malawi, with <10% of all contraceptive users relying on LAPMs (34, 35). One study in the capital of Malawi, Lilongwe, found that majority of the FP clinics did not offer IUD or female sterilisation services (35). In addition, relatively older women at the end of their reproductive years are often left out of FP discussions and policies due to the perception, sometimes wrongly, that they are menopausal, have infrequent sex, or lack a regular partner. Family planning messages and services are therefore rarely targeted at these women to satisfy their demand.

Among the adolescents (15–19 years) and older women (40 years and above) in this study, religious affiliation was the most significant predictor of mDFPS. About 68% of Anglican, CCAP, and Other Christian women had their demand for FP satisfied with modern methods compared to just 36% among Catholic, SDA, and Muslim women. This finding is consistent with the results of previous studies in Malawi (36–38). Overall, fundamental Catholic, Muslim, and conservative protestant denominations, such as the SDA, believe that it is God who controls the number of children women have. Therefore, modern contraceptive use is viewed as violating or interfering with God's law on procreation. In Malawi, even in urban areas, where upwards of 90% of women are affiliated with either Christianity or Islam, it is likely that such conservative religious views inform women's reproductive behaviour, especially contraceptive use. The fact that religion significantly predicted mDFPS among adolescents and older women suggests that religious constraints to modern contraceptive use are particularly severe among these already vulnerable and underserved groups.

## Limitations of the study

Even though this study is one of the few studies to examine mDFPS among urban women in sub-Saharan African using CHAID analysis, it has two key methodological limitations. Firstly, the study uses cross-sectional data from the 2015–16 Malawi Demographic and Health Survey which does not allow for causal inferences to be made from the findings. Secondly, the CHAID analytic technique used does not take into account the hierarchical structure of the Demographic and Health Survey data. In addition to these methodological limitations, the study did not include other factors such as the motivation for contraceptive use and fear of side effects which are known to determine women's capacity to satisfy their demand for FP with modern methods (39). Furthermore, the results of this study are limited to the Malawian context and representativeness of the 2015–16 DHS survey. Despite these limitations, the method used in this study revealed a complexity of interactions in the predictors of mDFPS that may be difficult to tease out in conventional regression analysis.

## Conclusion

This paper examined the predictors of mDFPS and identified the sub-groups of urban women in Malawi with unsatisfied mDFPS. The results showed that the number of living children, ethnicity, region of residence, exposure to FP information via text messages on phone, and exposure to FP information on TV were the significant predictors of mDFPS among women in urban Malawi. Among these factors, the number of living children a woman had was the most significant predictor of mDFPS. The findings also revealed that the sub-groups of urban women with unsatisfied mDFPS included women with no children who were not exposed to FP information/messages on TV; Ngoni, Yao, and other ethnic minority women who were exposed to FP information via text messages on phone; and Chewa, Lomwe, and Tumbuka women living in the northern and southern regions.

The disparities in mDFPS observed in this study demonstrate that the apparent urban advantage in modern contraceptive use in Malawi is not uniform across all sub-groups of women. There is therefore the need for policymakers and reproductive health practitioners to recognise these disparities and to prioritise the underserved groups identified in this study. As a matter of priority, policy and programmatic efforts in urban Malawi need to focus on women with no children, especially those without access to FP information on TV. Among women in union, adolescents, and women aged 40 years and above who are of Ngoni, Yao, and other minority ethnic groups, and who are also Catholic, SDA/Baptist or Muslim need to be prioritised as a matter of urgency. In addition to the policy implications, the study findings raised important and unexplored research questions such as the effect of exposure to FP information via text messages on mDFPS and the effect of partner's education on women's contraceptive uptake in urban Malawi. We therefore suggest further studies to address these research gaps.

## Data Availability Statement

Publicly available datasets were analysed in this study. This data can be found here: https://dhsprogram.com/data/.

## Ethics Statement

This study used data involving human participants, and was reviewed and approved by the National Health Sciences Research Committee in Malawi and ICF Institutional Review Board. Written Informed Consent was obtained from adult participants (18 years and above). For women below 18 years (15–17), consent to participate in the study was provided by their legal guardian in addition to the participant's own assent.

## Author Contributions

NA and NM conceptualised the study and decided on the data analysis. NA drafted the manuscript. Both authors contributed to the article and approved the submitted version.

## Conflict of Interest

The authors declare that the research was conducted in the absence of any commercial or financial relationships that could be construed as a potential conflict of interest.

## Abbreviations

mDFPS: Demand for Family Planning Satisfied with Modern Methods
CHAID: Chi-square (χ^2^) Automatic Interaction Detector.
